# Paths to cheminformatics: Q&A with Nathaniel Charest

**DOI:** 10.1186/s13321-023-00750-8

**Published:** 2023-10-05

**Authors:** Nathaniel Charest

**Affiliations:** Center for Computational Toxicology & Exposure, U.S. Environmental Protection Agency, Research Triangle Park, Durham, NC 27709 USA

## Introduction by the editors-in-Chief

Recently we described [1] an initiative to put a spotlight on diversity within the cheminformatics community. As part of that we initiated a series of interviews, and this article is part of that series.

Nathaniel Charest, Ph.D. (Chemistry), is a Chemist at the U.S. Environmental Protection Agency, where he develops in-silico and theoretical techniques to advance toxicological analysis and non-targeted identification of hazardous chemicals in the ecological milieu. He has expertise in cheminformatics, particularly in developing algorithms and tools for interactively exploring chemical space, automating and refining QSAR models of toxicological and physical endpoints, extracting insight and predictions from machine learning algorithms for integration into experimental design, quantifying and designing representative sets of chemically diverse libraries to assist non-targeted analysis experiments, and deep learning of latent features for chemical characterization, selection, and supervised prediction workflows.

## Interview questions

### Disclaimer

*These views do not reflect U.S. Environmental Protection Agency policy or opinions.*

### What has been your path to where you are today?

I grew up in Santa Barbara, California, where my deep family roots and various opportunities kept me throughout much of my life until finishing my Ph.D. My mother was a constant support in my life who kindled my interest in science by buying me a small microscope when I was 8, and always looking to help focus my interests. My mind always wandered, and my interests shifted, but she managed to hit the moving target and nurture my voracious curiosity. The concrete realization that I wanted a career in research was slow. But it began in high school when I discovered a stable affinity for physics, and this interest carried me into University of California Santa Barbara to study it formally.

Eventually, however, a passion for pharmacology changed my B.S. major to Biochemistry. I retained the quantitative focus by staying in close contact with the physics concepts that emerged through course material on protein conformations, thermodynamics, and the most mathematical elements of biochemical theory. After a very brief stint in an organic synthesis lab during which I realized I liked programming more than rotovapping, I had a conversation with my professor of thermodynamics, Dr. Joan-Emma Shea. I experience sharp embarrassment recalling how directionless I felt up until that moment Joan made it explicitly clear she thought I was suited for an advanced degree and am thankful to this day that she took the time to wade through my meandering thoughts on entropy and dynamics and help me crystalize my nebulous interests into something of value.

During my Ph.D. I never let go of information theory. There was something so mysterious and powerful about this thing that shows up everywhere – physics, chemistry, probability, signal processing. The quantification of information and knowledge itself is so deeply fascinating that it carried me into the literature of Shannon, Breiman, Hinton, and other visionaries of modern machine intelligence. While I think there were moments Joan questioned whether I was on track or just on a tangent, she always trusted me to work in the direction I wanted to. Ultimately, I was given the freedom to hybridize my love of machine learning and representation theory with the data generated by our molecular dynamics simulations, resulting in a bulk of doctoral work that I can fondly look back on.

When I had an opportunity to do contract work for the United States Environmental Protection Agency (EPA), the idealist in me felt the call. It was a massive shift – moving across the country – but Dr. Antony Williams was supportive and engaging, and he fully embraced my need to go into the mathematical weeds. I became his post doc, where I am happily working on the theory of quantitative structure-activity modeling and quantification of chemical spaces for non-targeted analysis.

### What is your current research focus, and what are your plans for the future?

My current research focus is divided into two primary domains. On one hand, I am pursuing the world of structure-activity modeling by trying to dive deep into how the algorithms we use interact with the molecular descriptors that are commonly employed. I am fascinated by the old graph theory and detailed ideas that were aggregated and, in some sad ways, forgotten by the literature during the machine learning revolution. I am so curious if the sort of careful, first-principles thinking we saw from Kier, Hall, Randić, and others from the prior century may have a role in a world increasingly ruled by bulk statistics and complex algorithms sometimes labeled, perhaps even dangerously, as black boxes beyond human comprehension.

The other research I do focuses on non-target analysis (NTA). This is an analytical chemistry field that, unlike the more common enterprise when one is trying to identify and characterize known substances in a sample, focuses on trying to identify and characterize the unknown substances in a sample. The potential utility of such technique is unbelievable, and at its current stage of evolution there is a lot of room for helping develop theoretical and computational tools for analytical chemists looking to take their measured signals and narrow down what compounds might possibly be generating them. Dr. Jon Sobus is a constant source of energy and motivation in this area, and I am thankful for the opportunity to apply my fascination with the abstract to a need that is so real and impactful.

### Which obstacles did you encounter during your career, and what experiences have helped you get where you are today?

I doubt I can speak to anything particularly spectacular or unique about my experiences. My own biggest obstacles were internal. My curiosity was a blessing and a curse, it was a need to learn but difficult to temper into something that could be called productive. I had to learn how to control my own impulse to run between fields, and I had to conquer the ubiquitous doubt that I was suited for anything. In other words, I was an undergraduate and then a graduate student.

More personally I have struggled at moments to communicate with the more cortisol-driven sect of the scientific community. I think sometimes the rush to succeed can overwrite the core tenants of our philosophy – interrogation, deconstruction, and patient ratiocination. And in our community, this can affect folks in subtle ways that creates unnecessary stress and dissuades some from joining the discourse. For instance, the lack of concrete appreciation for negative results, while almost universally accepted as a flaw in our academic and scientific culture, remains a burden on upcoming scientists who can already feel like failures in a field where the failure rate is, by construction, quite high. We are a group of highly intelligent humans, but we are, ultimately, all still human; the scientific method does not state every hypothesis must be proven correct to be of value.

I am blessed with a history of supportive family and teachers – Joan, Melissa Woods, Kalju Kahn, Irene Chen, and so many others stand out – who all helped shape my uncertainties into a research career that, today, I cannot imagine any alternative to. I am humbled by the fortune of having the stability and support in my life to grow into who I am today and must remain aware that my experience obligates me to try and, when I have that moment to make a difference, safeguard that the next generations have access to these conditions as well.

### What advice would you give your younger self?

The pedant in me always gets into the weeds of butterfly effects with this sort of question. At face value I would probably just tell him to relax and get some exercise more often. I think the modern term is ‘touch grass’. My old self would have really benefited from balancing time spent inside his head with time spent engaging the physical world.

Oh, and start yoga or anything flexibility related early. My lower back will thank you.

### What is a current challenge you are facing that should not be a challenge in the near future?

A current challenge I experience is balancing what would be state-of-the-art technological solutions with what is pragmatic. For instance, many researchers use workflows that require, for defensible reasons, spreadsheet structures of data management. However, today relational databases are the best practice for data management, and so frequently there is this question of how we are storing our data and how we can work with people not necessarily experts in data storage to best optimize organization-wide data management practices in a way that is both technologically optimal, but still accessible and tailored to the frontline scientists and regulators who utilize it. Similarly, the engineers who can implement these software best practices require feedback and input from the domain experts to help design their schemas and intuitively connect and organize all the data. There is a translation gap between the engineer universe and the science universe that can frustrate collaboration.

I suspect as better tools come into existence to interface these needs that the problem will alleviate.

### What do you think the cheminformatics community could do to increase diversity and inclusion?

I am not sure that, given my privilege, my insight into this is as honed as another might be, but I will do my best.

Cheminformatics is one of the most abstract fields in existence. For people to engage and pursue it, they must have satisfied enough basic needs to find a sense of security and spend time in their thoughts making cheminformatics real and meaningful to them. I believe if we could create these safe, happy conditions across impacted communities that, for historical, social, or economic reasons, have children struggling to feel stable and free, then the universal spirit of human curiosity and intellect will lead those communities to raise scientists who find fascination for the sheer coolness and beauty of cheminformatics. It is historical fact that if humanity had not learned to farm and establish stable food sources, then we would not have found time to send a space shuttle to the moon.

A part of the E.P.A.’s mission is the pursuit of Environmental Justice, and I believe initiatives like these will create a more stable, prosperous world for those socially or economically disadvantaged. This will yield social conditions where those impacted can step away from the struggle to satisfy their basic needs, and into a healthier space where they are free to develop skills and affection for our incredibly abstract field.

As a community of scientists, I think we must try to help ensure the technologies we facilitate lend themselves to the prosperity and safety of all communities and do our best to embrace nobility and generosity in moments where we can affect the fate of our work. Technology can push us into utopia or dystopia. We do not have all or even large influence over how what we create is used, but perhaps if we flap our wings at the right moment, we can push whatever happens toward prosperity for everyone, and in this indirect way build a future that is safe, diverse, just, and inclusive. Written in the E.P.A.’s atrium is Gaylord Nelson’s quote: *“*The ultimate test of man’s conscience may be his willingness to sacrifice something today for future generations whose words of thanks will not be heard.”

### What is your thought on ChatGPT/large language models and how these might influence the way we do science?

ChatGPT/LLM’s are tools. So are fire, metastable chemicals, Zippe centrifuges, halogenated polymers, and oil. There is a uniqueness to LLMs that they can present a facsimile of human intelligence, but in their current form I think it would be far more valuable for our community to first ask itself the philosophical question of how we will use these new tools rather than the technical question of what we will do with them.

I do worry that LLMs could accelerate a trend of outsourcing thought to algorithms. I have already expressed some consternation with the refocusing from the graph- and quantum- quantitative theory of the 20th century to the score-statistic dominated philosophy of the 21st century’s modeling. In the same way I am not comfortable if an analytical chemist cannot explain the mechanism leading from a chemical feature to their instrument’s signal, I tend to view with skepticism the notion that a signal generated by an algorithm is any less obligated to explain itself, or at least attempt to. I am at least inclined to exercise caution questioning whether a blob of linguistic probabilities is outputting defensible conclusions or may be vulnerable to the uncertainty and irreducible error in the data space that has been discussed by prior interviewees. There are already headlines about the ‘hallucinations’ being observed in natural language models where completely fabricated ‘thinking’ is passed off as unilaterally well-sourced. It seems clear that these models are far from perfect, and overutilizing these models too quickly could result in a reduction of information quality to that of the median quality of all information available on the internet.

### Photos


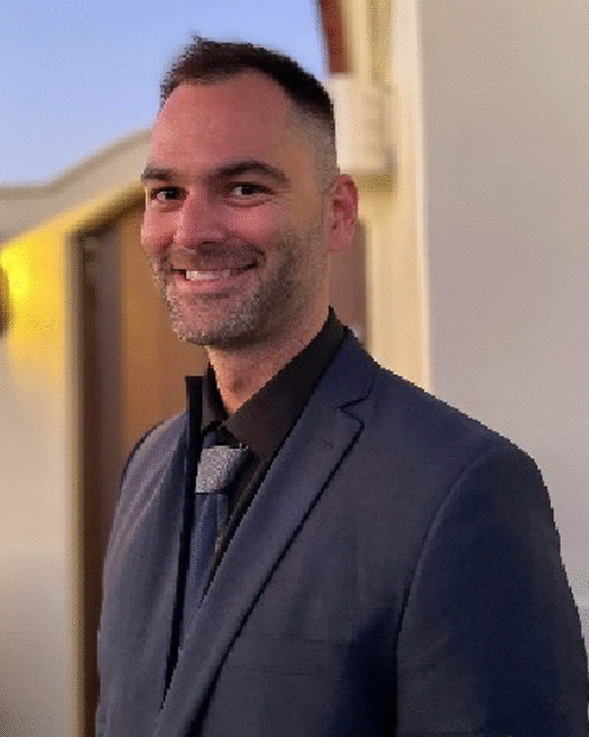


## Data Availability

Not applicable.
